# Otomycosis in Damascus, Syria: Etiology and clinical features

**DOI:** 10.29252/cmm.3.3.27

**Published:** 2017-09

**Authors:** Mohammad. T Ismail, Abeer Al-Kafri, Mazen Ismail

**Affiliations:** 1 Department of Microbiology, Faculty of Pharmacy, Arab International University (AIU), Ghabaghib, Daraa Governorate, Syria; 2 Research Department, Arab International University (AIU), Ghabaghib, Daraa Governorate, Syria

**Keywords:** *Aspergillus*, *Candida*, Otomycosis, Syria

## Abstract

**Background and Purpose:**

Otomycosis is a fungal infection that frequently involves the external auditory canal. The epidemiologic data on the etiologic agents of otomycosis in Syria are very limited. In this study, we aimed to determine the fungal agents, gender distribution, and clinical presentation of otomycosis.

**Materials and Methods:**

Two hundred and ninety nine patients (153 [51.17%] male and 146 [48.83%] female) clinically prediagnosed as otomycosis were studied at Al-mouassat University Hospital and ENT Crescent Syrian Clinic. Clinical samples were collected from the ear discharges and cultured on Sabouraud Agar.

**Results:**

Otomycosis was diagnosed in 70 (23.4%) cases, with the highest prevalence in males aged 16-75 years (73.6%). The isolation rates of mold and yeast fungi were 75.7% and 24.3%, respectively. The most common presentations were otorrhea (98.66%), otalgia (18.06%), and hearing loss (6.35%). Our results showed that 64.28% of otomycosis agents were *Aspergillus* species. *A.* *niger* was the most common agent (45.7%), and 24.3% of the pathogens were *C.* *albicans*.

**Conclusion:**

Otomycosis agents most commonly belonged to the genus of *Aspergillus* followed by *Candida*, which should be seriously considered by physicians for appropriate treatment.

## Introduction

Otomycosis is a fungal infection often located in the medial aspect of the external ear canal. This disease occurs as a primary infection or develops along with external bacterial infections as a result of antibiotic therapy (broad-spectrum antibiotics) [1, 2]. Otomycosis may be either subacute or acute and is characterized most commonly by ear itching (pruritus), discomfort, otalgia, malodorous discharge, otorrhea, scaling, sometimes hearing loss, and/or a feeling as if something is in the ear canal [2-4]. Otomycosis is treated with debridement followed by topical azole antifungals [5, 6] and symptomatically managed with oral antihistamines. Yaganeh in a study performed in Iran used 10 cc of 2% acetic acid plus 90 cc of 70% isopropyl alcohol, which was found to be effective [7].

The mycosis results in inflammation, superficial epithelial exfoliation, masses of debris containing hyphae, suppuration, and pain. The most common finding on ear examination is the presence of grayish white thick debris. *A.* *niger* and *C.* *albicans* are the most common responsible strains for fungal ear infections, which varies according to geographic location [8]. The incidence of otomycosis is higher in tropical and subtropical regions [2, 3], probably because of high ambient humidity, warmth, and darkness in the ear canal that promote fungal growth. Fungal organisms have a very characteristic appearance in the ear canal, especially under high magnification. Fungal filaments and spores may seem like mold growing on spoiled food. *A.* *niger* spores look like a fine coal dust sprinkled in the ear canal. Candidal infections typically are associated with soft, white, sebaceous-like material that may fill the ear canal in severe cases. A pseudomembrane often lines the ear canal that, when removed, reveals an underlying friable granular membrane [3]. 

There is no previously published study on this subject in Syria. Herein, we aimed to determine the prevalence of otomycosis and its causative agents.

## Materials and Methods

We enrolled 299 patients (153 males and 146 females; age range: 1-75 years) with clinical prediagnosis of otomycosis, who presented to Almouassat University Hospital and Syrian Arab Red Crescent clinic during February 2015-December 2016.

The scientific research council of Arab International University (AIU) approved the research study under the resolution number 6/4 dated on 23-3-2016. Clinical details such as chief complaint, name, age, gender, suspected risk factors, history of infection, address, and other relevant information were recorded. Informed written consent was obtained from all the patients. Clinical samples were collected by swabs of ear discharge, the color of which ranged from white to black. Afterwards, the samples were cultured on Sabouraud Dextrose Agar with chloramphenicol (SDA, Difco) and Sabouraud Dextrose Agar with chloramphenicol and actidione (cycloheximide) (SCC; AVONCHEM, ACM-2460, Wellington House, UK), then incubated at 25°C for up to seven days. Fungal identification was based on colony morphology and microscopic examination of fungal structure, whereas germ tube test was utilized for the identification of *Candida albicans*. Cultures were examined daily up to seven days to determine the probable growth of fungal colonies and their identification. The identification process of the isolated fungi was carried out via the traditional methods [9]. Cultures were prepared and examined microscopically. The fungi were identified based on the conventional methods.

The data were analyzed using Medcalc for windows, version 14 (Medcalc software, Ostend, Belgium). Percentages and 95% confidence interval were used to perform the exploratory analysis of the categorical variables, while quantitative variables were presented as mean±standard deviation. Pearson correlation coefficient, Chi-squared test, and Fisher's exact test were used to explore the relationship between categorical data. *P-value* less than 0.05 were considered statistically significant.

## Results and Discussion

In otomycosis, fungi may cause either primary or secondary invasion after tissue abnormality resulting from a primary bacterial infection. Therefore, otomycosis can be observed in mixed bacterial-fungal infections [4, 10, 11]. The main risk factors for otomycosis include moisture, minor inflammation, the use of broad-spectrum antibiotics, steroids, chemotherapeutic agents, topical ear drops, physical injury, living in warm and humid climates, and frequent bathing or swimming [12, 13].

The epidemiologic data on the etiologic agents of otomycosis in Syria are limited, therefore; we purported to investigate the agents of otomycosis in patients living in Damascus, Syria. Otomycosis studies in patients with suspected mycosis have been conducted in many different countries, including Iran [8], the USA [14], Spain [15], Turkey [16], Nigeria [17], and Nepal [18]. The results of these studies suggest that otomycosis has a global prevalence. 

The most common presentations in our study were otorrhea (295; 98.66%, CI: 262.3 to 330.6), followed by otalgia (54; 18.06%, CI: 10.7 to 28.4), and hearing loss (19; 6.35%, CI: 2.8 to 14.4). However, in another study, the most common presenting symptom was hearing loss (77.7%), followed by pruritus (68.8%) and otalgia (40%) [19].

There were 299 patients with documented diagnosis of otomycosis, 153 (51.2%; CI: 129.7 to 179.3) of whom were males and 146 (48.8%; CI: 123.2 to 171.7) were females. There was no significant relationship between gender and the isolated fungi (*P=0.9160*).

The age of patients ranged between 1 and 75 years with the mean age of 33.1±22.27 (95% CI: 30.54- 35.61) years.

From 299 samples only 70 cases (23.4%) had growth on both media culture SC and SCA which indicate that the nature of these fungi in ear canal were pathogenic. 

In our study, the prevalence of otomycosis was 23.4%, which is lower than the results found in some other studies including the work by Kumar [20], who found otomycosis in 75.9% of patients. Pardhan et al. [18] observed it in 79.4% of patients, while Kazemi et al. [21] reported otomycosis in 92% of patients. This condition was detected in 96.43% 64.8%, 39.6%, and 88.6% of the cases in the studies by Kiakojuri et al. [22], Morozova et al. [23], Fayemiwo et al. [24], and Agarwa et al. [2], respectively. Our results were in line with those reported by Degerli et al. [25], who reported this disease in 24% of patients.

The isolation rates of molds and yeasts (*C.* *albicans*) were 53/70 (75.7%) and 17/70 (24.3%), respectively. The relationship between gender and types of isolated fungi was not significant (*P=0.23*). *A.* *niger* was the most common isolated mold (32/70; 45.7%), followed by *A.* *versicolor* (10/70; 14.3%), *Penicillium *spp. (8/70; 11.4%), *A.* *fumigatus* (2/70; 2.9 %), and *A. flavus* (1/70; 1.4%; Table 1).

*A*.* niger* and *C. albicans* were found the most prevalent fungi in our study. Similar results were obtained by other studies [26-28]. Our results showed that 64.28% of otomycosis agents belonged to the *Aspergillus* genus. Similar results were reported by Kiakojuri et al. [22] and Saki et al. [29]. In an Iranian study, the prevalence rate of *Aspergillus* genus was 78.59% [1]. There was no significant association between gender and the isolated *Aspergillus* species (*P=0.32*).

**Table:** **Table 1**Fungal species isolated from the cases of otomycosis

**Positive samples**	***Candida*** ***albicans***	***Aspergillus*** ** species**				***Penicillium*** ** spp.**
		***Aspergillus*** ***niger***	***Aspergillus*** ***versicolor***	***Aspergillus*** ***fumigatus***	***Aspergillus*** ***flavus***	
70	17	32	10	2	1	8
100%	24.3%	45.7%	14.3%	2.9%	1.4%	11.4%
95% CI	11.4 to 29.6	21.8 to 45.1	4.8 to 18.4	0.2 to 7.2	0.02 to 5.5	3.4 to 15.7

*A.* *niger* was the most common mold isolated in our study (45.7%). Similar results were obtained in Turkey [25], Iran [8, 21, 22] and Egypt [30]. In a study conducted in Ibadan, the rate of *A.* *niger *infection was very low (1.9%) [24]. Further, the rate of *A.* *versicolor* was 14.3%, while in a Nigerian study by Itah et al. [31], this rate was 30% among the fungi responsible for otitis. 

In our study, the rate of *A.* *fumigates* infection was 2.9%, while its prevalence rates in the USA [32], Turkey [25], Nigeria [24], India [2] and Iran were 13%, 26.46%, 5.7%, 11.9%, and 5.35%, respectively [22]. The prevalence of *A.* *flavus* in our study was 1.4%, while in Iran it was 3.57% [22] and in an Indian study it was 19.9% [2].

The incidence rate of otomycosis due to *C.* *albicans* in our study was 24.3%; the link between gender and isolated *Candida* was not significant (*P=0.91*). A similar result was obtained in Ibadan, Nigeria (28.3%) [24]. Several studies published in Iran showed that the incidence rate of otomycosis due to *Candida* spp. ranged from 7% to 19.64% [8, 21, 22], while in India [2] and the USA [32], this rate was 10% and 43%, respectively. This discrepancy in results could be attributed to geographical variations. *Penicillium* spp. infection rate in our study was 11.4%, which is higher than the reports from Ibadan (1.9%) [24], India (1.4%) [2], and Iran (1.79%) [22].

In conclusion, it is notable that the most common causative agent of otomycosis in Syria was *Aspergillus*, particularly *A.* *niger*, followed by *C.* *albicans*. These fungi are pathogenic because they grow on SCA and patients recover after antifungal treatment. Same results have been established by many studies around the globe [22, 26-28, 33], but some studies have shown many other causative fungal species of otomycosis. These agents include species from the genera *Fusarium*, *Mucoraceae*, *Scopulariopsis*, *Alternaria*, *Malassezia*, *Rhizopus*, *Cladosporium*, *Geotrichum*, and various dermatophytes [2, 15, 22, 25, 34]. 

This study stresses on the importance of sampling from ear canal lesions in patients with otitis to provide proper diagnosis and treatment.
